# Cross-contamination of lettuce with *Campylobacter* spp. via cooking salt during handling raw poultry

**DOI:** 10.1371/journal.pone.0250980

**Published:** 2021-05-19

**Authors:** Nânci Santos-Ferreira, Ângela Alves, Maria João Cardoso, Solveig Langsrud, Ana Rita Malheiro, Rui Fernandes, Rui Maia, Mónica Truninger, Luís Junqueira, Anca Ioana Nicolau, Loredana Dumitrașcu, Silje Elisabeth Skuland, Gyula Kasza, Tekla Izsó, Vânia Ferreira, Paula Teixeira

**Affiliations:** 1 Universidade Católica Portuguesa, CBQF—Centro de Biotecnologia e Química Fina–Laboratório Associado, Escola Superior de Biotecnologia, Porto, Portugal; 2 Nofima, Norwegian Institute of Food, Fisheries and Aquaculture Research, Ås, Norway; 3 Histology and Electron Microscopy (HEMS), Instituto de Investigação e Inovação em Saúde—i3S, Instituto de Biologia Molecular e Celular—IBMC e Universidade do Porto, Porto, Portugal; 4 UFP Energy, Environment and Health Research Unit (FP-ENAS), Universidade Fernando Pessoa, Porto, Portugal; 5 Universidade de Lisboa, Instituto de Ciências Sociais, Lisboa, Portugal; 6 Faculty of Food Science and Engineering, Dunărea de Jos University of Galați, Galați, Romania; 7 Consumption Research Norway (SIFO), Oslo Metropolitan University, Oslo, Norway; 8 Department of Risk Prevention and Education, National Food Chain Safety Office, Budapest, Hungary; University of Patras, GREECE

## Abstract

C*ampylobacter* spp. are the most common bacterial pathogens associated with human gastroenteritis in industrialized countries. Contaminated chicken is the food vehicle associated with the majority of reported cases of campylobacteriosis, either by the consumption of undercooked meat or via cross- contamination of ready-to-eat (RTE) foods during the handling of contaminated raw chicken parts and carcasses. Our results indicate that cooking salt (used for seasoning) is a potential vehicle for *Campylobacter* spp. cross-contamination from raw chicken to lettuce, through unwashed hands after handling contaminated chicken. Cross-contamination events were observed even when the chicken skin was contaminated with low levels of *Campylobacter* spp. (ca. 1.48 Log CFU/g). The pathogen was recovered from seasoned lettuce samples when raw chicken was contaminated with levels ≥ 2.34 Log CFU/g. We also demonstrated that, once introduced into cooking salt, *Campylobacter* spp. are able to survive in a culturable state up to 4 hours. After six hours, although not detected following an enrichment period in culture medium, intact cells were observed by transmission electron microscopy. These findings reveal a “novel” indirect cross-contamination route of *Campylobacter* in domestic settings, and a putative contamination source to RTE foods that are seasoned with salt, that might occur if basic food hygiene practices are not adopted by consumers when preparing and cooking poultry dishes.

## Introduction

*Campylobacter* is the major cause of bacterial diarrheal illness worldwide, and the bacterial agent that most contributes to the global burden and economic costs of foodborne illnesses [1, 2]. *Campylobacter* infections (campylobacteriosis) frequently result in self‐limiting mild gastroenteritis. However, it may develop into a severe illness, including Guillain–Barré syndrome, reactive arthritis, bacteremia, or even death; particularly among the very young, elderly, and immunosuppressed individuals [3]. Among the many species and subspecies assigned to the genus *Campylobacter*, *Campylobacter jejuni* (subspecies *jejuni*) and *Campylobacter coli*, account for the majority of the human cases reported [2], and in less extent, *Campylobacter lari*, *Campylobacter fetus*, and *Campylobacter upsaliensis* [4, 5].

*Campylobacter* spp. colonize the gastrointestinal tract of several animals however, poultry species are the most common hosts for *Campylobacter* and are recognized as the main source of human infection [5]. Generally, the bacterium colonizes the cecum and colon of poultry flocks, particularly chickens, in high levels, resulting in carcass and meat contamination after slaughter, during defeathering and evisceration [6]. Due to its fastidious growth requirements, temperatures above 30°C and micro-aerobic conditions, growth of *Campylobacter* is significantly limited during processing and storage of poultry meat [5, 7].

Regarding human exposure to *Campylobacter* due to contaminated poultry, epidemiological studies show that two important pathways exist: eating undercooked poultry meat and cross-contamination events [8–12]. As the average prevalence of *Campylobacter* on the surface of poultry is higher (62.3%) in relation to its prevalence inside the poultry meat (10.3%) [13], and presuming that cooking procedures will inactivate most of the bacteria present on the surface, the contribution of cross-contamination events to consumers’ exposure to *Campylobacter* infections is assumed to be more relevant than undercooking [13–15]. A variety of cross-contamination scenarios and routes of contamination in the food preparation environment have been extensively investigated, both in simulated laboratory experiments and in private domestic kitchens, including the transfer rates from raw poultry into cutting boards, cutlery, plates, or kitchen surfaces and from these items to RTE foods [16–22]. Unwashed hands after raw handling poultry has also been established as an important vehicle of *Campylobacter* cross-contamination of RTE food, or food contact surfaces. The majority of the studies on this subject report that the consumer style hand-washing procedures are ineffective in preventing the transfer of *Campylobacter* spp. [16, 22–25], even when soap is used [22], indicating that consumers should be advised to avoid hand contact with raw poultry and to improve their hand-washing procedures.

A qualitative consumer observational study and a cross-national quantitative consumer survey (both previously conducted in the scope of the SafeConsume project (http://safeconsume.eu) were the starting point for this study. The observational study was conducted in six European countries (France, Hungary, Norway, Portugal, Romania, United Kingdom), in consumers’ private kitchens during the preparation of a recipe that included chicken and a raw vegetable salad according to the methodology described by Skuland et al. [26]. The quantitative consumer survey was conducted across ten European countries (Denmark, France, Germany, Greece, Hungary, Norway, Portugal, Romania, Spain, United Kingdom) to allow the measurement of problematic food handling behaviour in a standardised, quantitative and cross-nationally comparable manner. Both studies revealed several possibilities of cross-contamination of salads linked to raw poultry handling practices [26; S1-S3 Tables in S1 File]. With the exception of the Norwegians, several consumers from the other countries store cooking salt in bowls, use the same salt to season chicken and RTE salads and this may be done using their bare hands (S4 Table in S1 File). After a careful literature search, we realized that cooking salt was never referred or investigated as a potential vehicle of cross-contamination of RTE foods. In this context, this study aimed to investigate the potential role of cooking salt as a vehicle of indirect cross-contamination of vegetable salads with *Campylobacter* spp. during handling of raw chicken. A route of cross-contamination was designed, to simulate a domestic meal preparation scenario. The transfer of campylobacters from inoculated chicken meat onto cooking salt and then to lettuce was evaluated.

## Materials and methods

### Bacterial strains and inoculum preparation

A mixture of eight *Campylobacter* spp. strains was used for inoculations in all assays, including six *C*. *jejuni* (DSM 4688, DFVF 1099, NCTC 11168, CJ305, C9, C21A) and two *C*. *coli* (DSM 4689 and C3) (S5 Table in S1 File). Strains were preserved at -80°C in cryovials containing brain heart infusion broth (BHI, Biokar Diagnostics, France) supplemented with 20% (vol/vol) glycerol and 5% of lysed defibrinated horse blood (bioMérieux, France). To prepare inocula, strains were streaked onto Columbia agar supplemented with 5% (v/v) of lysed defibrinated horse blood (Columbia blood agar, bioMérieux) and incubated at 41.5°C under microaerophilic atmosphere (5% CO_2_; Heracell CO_2_ incubator, Heraeus, Germany) for 48 h. Subsequently, colonies were harvested to prepare a cell suspension for each strain in 1/4 strength Ringer solution (Biokar Diagnostics) adjusted to an OD_600_ = 1, corresponding to ca. 10^8^ CFU/mL. A cocktail containing all strains was prepared by combining equal volumes of individual cell suspensions in a sterile test tube. Decimal dilutions were further performed in Ringer solution to obtain mixed culture inocula with different bacterial levels to be used in experiments detailed below; in addition, 0.1 mL aliquots of appropriate dilutions were spread-plated on Columbia agar enriched with blood, in duplicate, to ascertain the actual CFU/mL present in the inocula.

### Cross-contamination experiment

**Fig 1:**
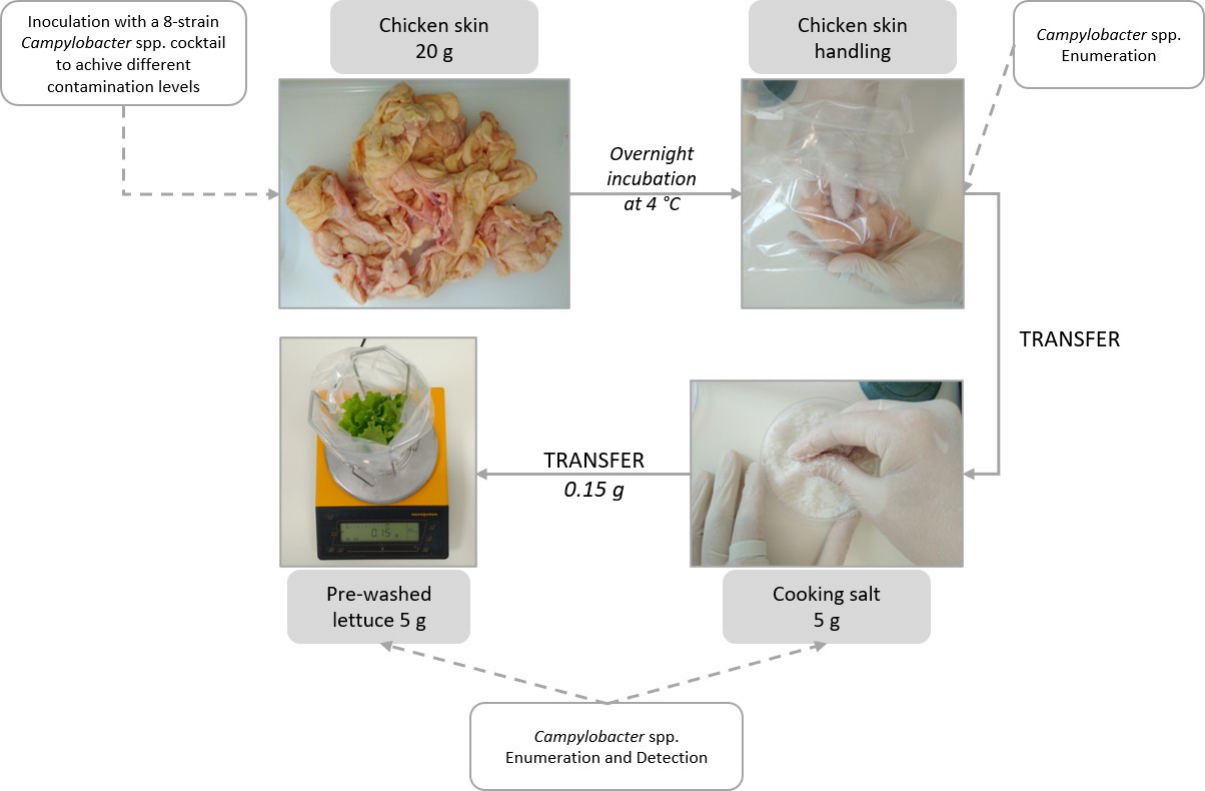
Model of cross-contamination with *Campylobacter* spp. from artificially inoculated chicken skin to RTE lettuce via cooking salt.

A model of *Campylobacter* cross-contamination from raw chicken skin to RTE salads via cooking salt was designed and tested (Fig 1).

Entire chicken (*Gallus gallus domesticus*) carcasses (one chicken carcass for each trial), fresh lettuce and purified sea salt in big white crystals (97.7% NaCl; designated from now on as cooking salt) were purchased from a local supermarket. All the poultry preparation material (including cutting boards, knives, and scissors) was sterilized with 70% ethanol or alcohol flame sterilized (with 96% ethanol) before use. For each chicken carcass, all the skin was aseptically removed cut into small pieces and manually mixed. 20 g samples were placed in sterile stomacher bags (BagLight PolySilk, Interscience, France) to be used in the transfer experiments, including two non-inoculated control samples to be used in the enumeration and detection of naturally occurring *Campylobacter* spp. Poultry skin samples were inoculated with 1 mL of five different inoculum levels of *Campylobacter* mixtures in order to achieve final contamination levels in the skin ranging from 10^1^ to 10^5^ CFU/g. The skin samples were then manually homogenized until total absorption of the cell suspension to ensure an equal distribution over the skin surface. Samples were stored overnight at 4°C to mimic retail and/or household storage conditions and to promote bacterial attachment. Immediately before cross-contamination assays, 5 g of cooking salt were weighted into Petri dishes using an alcohol flame sterilized spoon, and 5 g portions of lettuce leaves, previously washed with running tap water and air dried, were aseptically weighed into stomacher bags. The transfer experiments were performed by one volunteer using powder free latex gloves (VWR Chemicals, Belgium). The volunteer was instructed to handle/touch the chicken skin inoculated with *Campylobacter* and then stir the cooking salt in the Petri dish with the hand that touched the skin. Immediately after, the salt was homogenized with a sterile spatula and 0.15 g, the equivalent of 1/2 teaspoon of salt (ca. 3g) to season 100 g of lettuce, were scattered over the lettuce leaves and left undisturbed for 10 min at room temperature. *Campylobacter* detection and enumeration was performed as described below to determine contamination levels in the remaining cooking salt (ca. 4.85 g) and lettuce. Levels of *Campylobacter* naturally occurring on the skin samples used in each assay and in inoculated chicken skin samples after storage at 4°C and before the experiments were determined.

Three independent trials, i.e., performed in different days and by different volunteers, with duplicates per each initial skin contamination level, were carried-out, in a total of 30 cross-contamination events. Gloves were changed on each transfer assay. Uninoculated samples of cooking salt and lettuce were also included to rule out possible natural contamination by *Campylobacter*.

### Survival experiment in cooking salt

Five grams of cooking salt were aseptically weighed into sterile Petri dishes using an alcohol flame sterilized spoon and inoculated with five drops of 20 μL of the *Campylobacter* cocktail, prepared as described above (bacterial strains and inoculum preparation), randomly distributed over the salt surface, to reach a final level of ca. 10^5^ CFU/g. The Petri dishes were maintained at room temperature during the experimental period to mimic storage of cooking salt in consumer’s kitchens. The number of colony forming *Campylobacter* was determined (as described below) before inoculation and at predetermined time intervals: 0 (i.e. immediately after inoculation), 10, 20, 30, 45, 60, 90, 120, 150, 180, and 240 min. In addition, at time 120, 150, 180 min, and 240 min, the presence/absence of *Campylobacter* in 5 g of salt, i.e., was also assessed. These sampling times were established on the basis of results from preliminary experiments showing that viable cells could be recovered for four hours, with a marked loss of viability in the first few minutes in contact with the salt. The survival assays were carried out in four independent experiments. During the experimental time period, as a control for *Campylobacter* survival, 0.1 mL aliquots of the *Campylobacter* cocktail were deposited directly on the sterile Petri dishes, and at regular intervals, sample volume was collected and the Petri dish was rinsed with buffered peptone water (BPW; Biokar Diagnostics) for CFU/mL determination.

### Enumeration and detection of *Campylobacter*

Enumeration and detection of *Campylobacter* spp. were performed following, respectively, ISO 10272–2:2017 [27] and ISO 10272–1:2017 [28] recommendation with minor modifications.

In the transfer assays, 20 g of chicken skin and 5 g of cooking salt or lettuce were placed into a sterile stomacher bag and homogenized with 180 mL or 45 mL (respectively) of BPW for 2 min (BagMixer S, Interscience, France). In the survival assays, at predetermined time intervals, the 5 g of cooking salt were placed into a sterile stomacher bag and manually homogenized with 45 mL of sterile deionized water until dissolution of the salt was complete. The homogenates were serially diluted (1:10) in 9 mL of Ringer solution, and 1 mL of the first decimal dilution (distributed on the surface of three agar plates) or 0.1 mL aliquots of the further decimal dilutions, were spread-plated onto selective agar plates (modified charcoal cefoperazone deoxycholate (mCCD); VWR Chemicals, Belgium) and incubated for 44 h at 41.5°C in microaerophilic conditions. The detection limit of the enumeration method was 10 CFU/g.

Detection of *Campylobacter* spp. was performed for all lettuce and salt samples of the transfer assays, while in the survival on artificially contaminated cooking salt experiments, *Campylobacter* detection was only performed at times 120, 180 and 240 min. Briefly, the salt homogenates prepared for *Campylobacter* enumeration were filtered through a 0.45 μm pore size cellulose membrane filter (Frilabo, Portugal) and the membrane was placed into 45 mL of Bolton Broth (BB plus selective supplement; VWR Chemicals, Belgium) containing 5% of lysed defibrinated horse blood (Oxoid, UK). In lettuce samples homogenates it was not possible to preform filtration due to clogging of the filter membrane with small fragments of lettuce. Therefore, in this case, 5% of lysed defibrinated horse blood and 250 μL of Bolton broth selective supplement (VWR Chemicals, Belgium) were added to BPW, as this method has been reported to perform similarly as Bolton broth in isolation of *C*. *coli* and *C*. *jejuni* [29]. Enrichment bags were incubated at 41.5°C, for 44 h. After incubation, 10 μL of the enriched sample was transferred onto selective agar mCCD and CampyFood agar (CFA, bioMérieux) and incubated for 44 h at 41.5°C for qualitative analysis. The results were expressed as presence (growth) or absence (no growth) of *Campylobacter* in 5 g of lettuce or salt, or in 20 g of chicken skin. Quantification and detection of *Campylobacter* levels in 20 g of non-inoculated chicken skin used for the transfer assays were also performed to evaluate naturally occurring *Campylobacter* in these samples.

### Confirmation of *Campylobacter* species

In lettuce and salt samples, as uninoculated controls were *Campylobacter* free, typical colonies of *Campylobacter* on selective agar media obtained from detection and/or enumeration methods were assumed as *Campylobacter* spp. without further confirmation. On the other hand, in the chicken skin, both in inoculated and control (uninoculated) samples, up to five typical colonies of each selective agar plate were sub-cultured on Columbia agar (Merck Millipore, Massachusetts, United States) supplemented with 5% defibrinated horse blood and incubated under microaerophilic conditions for 24 h for further confirmation, including observation of haemolysis after 24h incubation, microscopy of a freshly prepared bacterial suspension, oxidase test and growth under aerobic conditions [27, 28].

### Transmission electron microscopy

*Campylobacter* cocktail morphology immediately after inoculation and at time 60, 150, and 360 min in cooking salt were analyzed by transmission electron microscopy (TEM). Samples were fixed overnight with 2.5% glutaraldehyde/2% paraformaldehyde in cacodylate buffer 0.1 M (pH 7.4). Samples were washed in 0.1 M sodium cacodylate buffer and fixed in 2% osmium tetroxide in the 0.1 M sodium cacodylate buffer overnight, followed by new fixation in 1% uranyl acetate overnight. Dehydration was performed in gradient series of ethanol solutions and propylene oxide and included in Epon resin by immersion of samples in increasing series of propylene oxide to EPON (till 0:1 ratio) for 60 min each. Sample inclusion in EPON resin was performed in a silicon mould. Sections with 60 nm thickness were prepared on a RMC Ultramicrotome (PowerTome, USA) using a diamond knife and recovered to 200 mesh Formvar Ni-grids, followed by 2% uranyl acetate and saturated lead citrate solution. Visualization was performed at 80 kV in a (JEOL JEM 1400 microscope (Japan)) and digital images were acquired using a CCD digital camera Orious 1100 W (Tokyo, Japan), at the HEMS core facility at i3S, University of Porto, Portugal.

## Results

### Cross-contamination of *Campylobacter* spp. from chicken skin to cooking salt, and from cooking salt to lettuce

**Fig 2:**
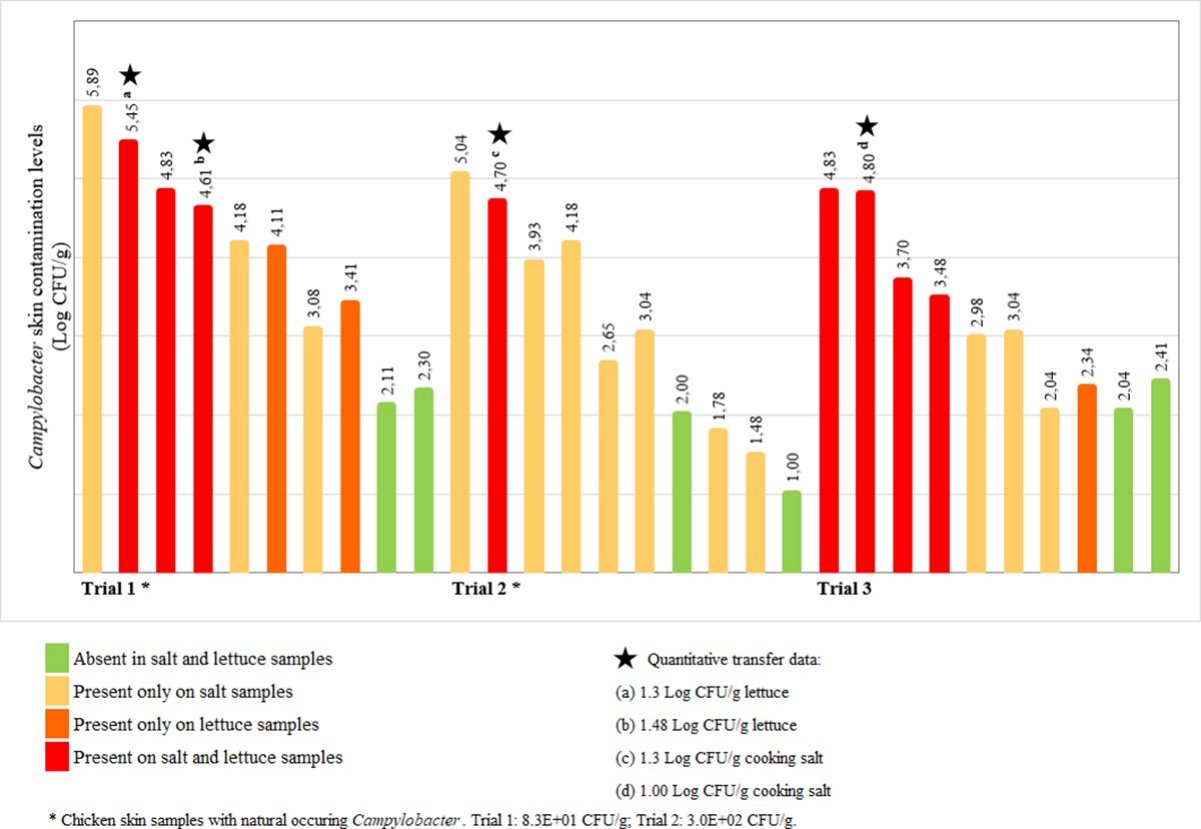
Transfer of *Campylobacter* from the contaminated chicken skin to cooking salt via unwashed hands, and from cooking salt to lettuce, according to the different initial skin surface contamination levels. The presence/absence (qualitative transfer data) of the pathogen on salt and lettuce samples is indicated by the different colours. Quantitative transfer data (i. e. numbers of *Campylobacter* organisms) was achieved in four samples (a) 1.30 Log CFU/g lettuce; (b) 1.48 Log CFU/g lettuce; (c) 1.30 Log CFU/g cooking salt; (d) 1.00 Log CFU/g cooking salt.

*Campylobacter* naturally occurring on the skin samples used in each assay varied between <10 CFU/g and 3.0x10^2^ CFU/g. After inoculation of the chicken skin samples with an 8-strain cocktail of *Campylobacter* spp. at different concentrations, final contamination levels ranging from 1.00 to 5.89 Log_10_ CFU/g were achieved (Fig 2). Naturally occurring *Campylobacter* in salt and lettuce was below the detection limit (10 CFU/g) in all samples before artificial inoculation.

In total, cross-contamination was detected in 24 out of the 30 events (80%) even for initial contamination levels in chicken skin as low as 1.48 Log CFU/g. For the remaining six events (20%) on which *Campylobacter* was not detected in either salt or lettuce skin samples were contaminated with values always lower or equal to 2.41 Log CFU/g (Fig 2).

Cross-contamination from chicken to cooking salt was observed in 21 events. Enumeration of *Campylobacter* was possible in two of the contaminated salt samples. Levels of 1.30 Log CFU/g and 1.00 Log CFU/g of salt were detected when the contamination of chicken skin was, respectively, 4.70 and 4.80 Log CFU/g. On the remaining samples *Campylobacter* was present but in levels < 10 CFU/g (Fig 2).

Eleven out of 30 events (37%) resulted in *Campylobacter* transfer from the chicken skin to lettuce, through seasoning with cooking salt. In three of the lettuce samples, *Campylobacter* was detected although the respective salt samples were negative. Enumeration of *Campylobacter* was possible in two lettuce samples at levels of 1.30 Log CFU/g and 1.48 Log CFU/g when the contamination of chicken skin was 5.45 and 4.61 Log CFU/g, respectively (Fig 2).

### Survival of *Campylobacter* spp. in experimentally contaminated cooking salt

**Fig 3:**
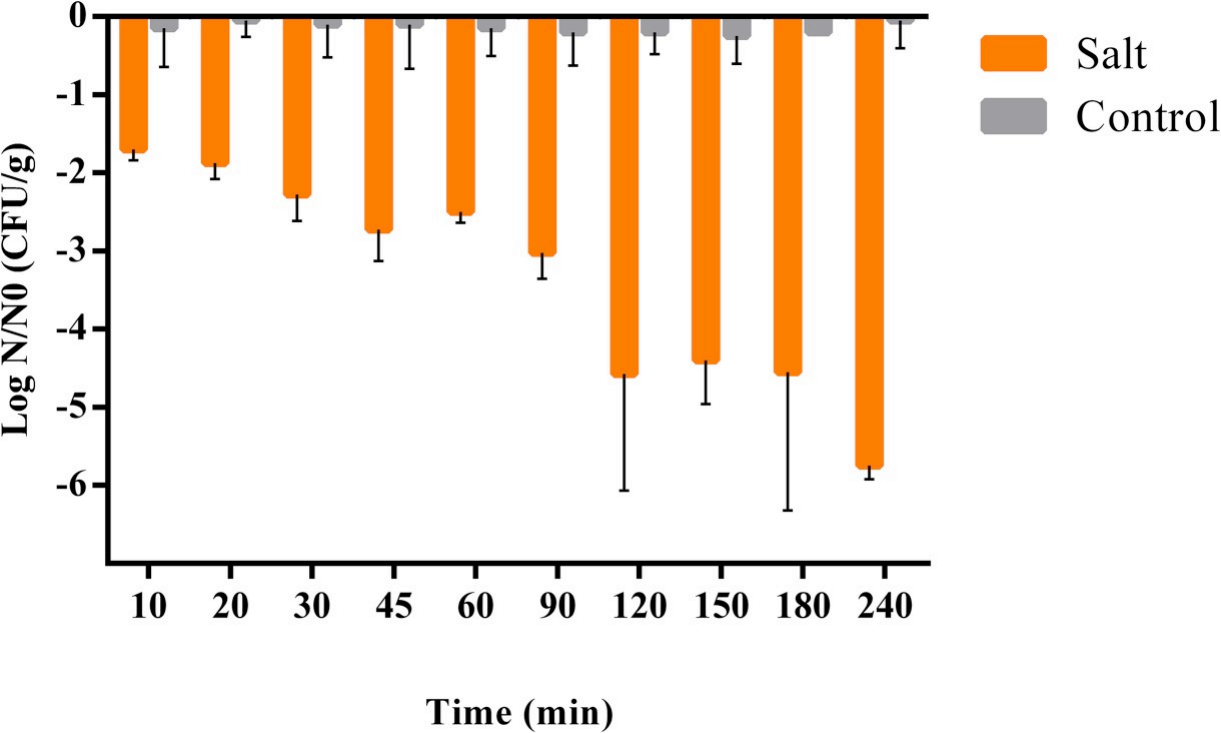
Survival of *Campylobacter* spp. in cooking salt. Results are presented as survival/difference from the control calculated as Log (N/N_0_) (where *N*_*0*_ is the bacterial number in the controls at time zero, and *N i*s the bacterial number at different times after inoculation). Columns present mean and standard deviation of four independent experiments.

Survival of *Campylobacter* cocktail in cooking salt was evaluated for 240 min. Logarithmic reduction of N/N_0_ CFU/g for all times studied are presented in Fig 3.

*Campylobacter* spp. survived in cooking salt up to 4 hours, as determined by culture-dependent methods. In control conditions, without exposure to salt, the number of viable cells remained constant (Fig 3). Reductions ranging from 1.7- to 2.3- log cycles were observed in cell survival in the first 30 min of exposure. After 1 h of exposure the cells viability was reduced 2.5- log cycles. Inactivation of *Campylobacter* to levels below the detection limit of the method (< 10 CFU/g) was variable among experiments, and observed either at 120, 180 min or at 240 min. On seven occasions, although viable counts were below 10 CFU/mL, *Campylobacter* was detectable after enrichment in Bolton, namely at sampling times 120 min (n = 1 and n = 2), 180 min (n = 1, n = 2 and n = 3) and 240 min (n = 3 and n = 4). On two occasions at sampling time 240 min, *Campylobacter* was not detected after enrichment.

### Transmission electron microscopy

**Fig 4:**
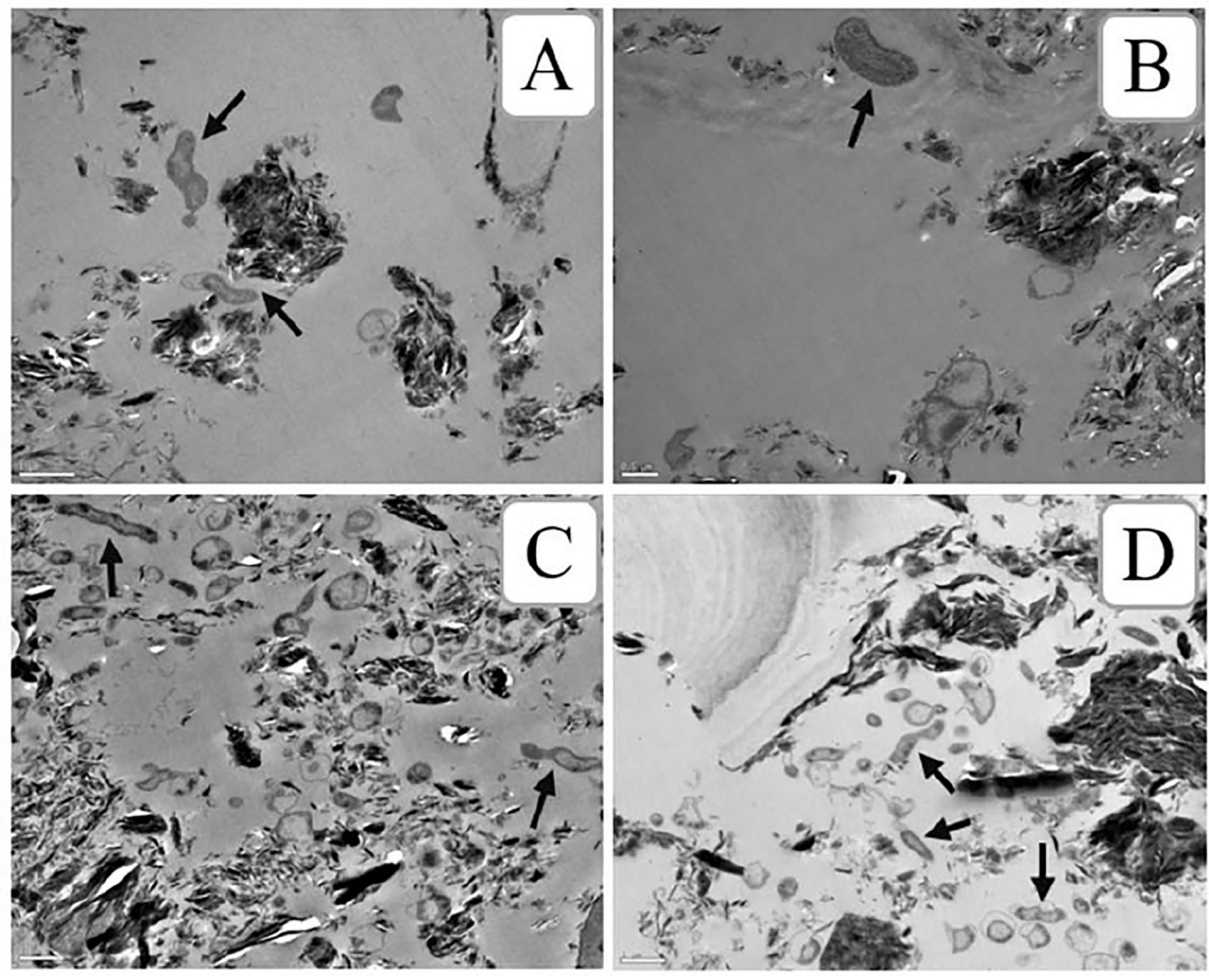
Transmission electron micrographs of cells of *Campylobacter* spp. after exposure to cooking salt at different time intervals showed spiral shaped cells even after 6 hours of exposure. A) immediately after inoculation; B, 60 min; C, 180 min; D; 6 hours. Arrows represent bacterial cells with typical spiral shape of *Campylobacter* spp. Amplifications: A) 15000X; B) 20000X; C) 12000X; and D) 12000X. Bar represents 1 μm in Fig 4A), 4C) and 4D) and 0.5 μm in Fig 4B).

The morphology of *Campylobacter* cocktail inoculated on cooking salt was observed by TEM immediately after inoculation and at time 60, 150 and 360-min of exposure (Fig 4). Due to the high level of impurities in cooking salt, it was not possible to do any quantitative assessment and provide an overall picture at the population using TEM images. Nevertheless, for all the times analysed, cells with an integral bacterial structure and maintaining their characteristic spiral shape were observed.

## Discussion

As previously mentioned in the introduction, consumers typically touch the raw chicken with their bare hands when preparing it [26] and a large majority of consumers do not wash their hands properly after handling poultry [26, 30, 31]. In this study we aimed to test the hypothesis that salt, contaminated during the handling of raw chicken, can be a source of contamination of ready-to-eat salads. Although according to popular wisdom "salt kills everything" and may be used to justify some unsafe practices, to our knowledge there are no published studies on the persistence of bacterial foodborne pathogens in this matrix. For the experiments conducted in this study the inocula were prepared from isolated colonies grown on agar plates and resuspended in buffered solution to avoid culture medium debris that could interfere with the pathogen survival in the inoculated salt. Furthermore, we used small volumes of inocula (100 μL) and carefully dispersed in small drops (20 μL), to contaminate the 5 g of salt samples. Nevertheless, it is important to consider that in a realistic scenario raw poultry meat contain natural juices that would be transferred to the cook hands and then to the cooking salt jointly with the *Campylobacter* cells, and possibly facilitate the pathogen survival in such harsh environmental conditions.

There is not much information available on *Campylobacter* numbers occurring in different poultry parts and carcasses but previous studies demonstrated that higher counts are detected on the surface than in the interior and that different levels are found in different chicken parts [13]. Levels of contamination are generally low although counts higher than 10^5^ CFU/g have been reported [13, 32]. In this study we tested *Campylobacter* contamination levels in the chicken skin in the range of values reported for chicken meat available in the market. Our results demonstrate that the transfer events take place randomly, therefore it is not possible to specify an acceptable risk level, e.g. salt and lettuce samples were free of *Campylobacter* when the initial counts on chicken skin was 2.41 CFU/g, while chicken skins with 1.48 and 2.34 Log CFU resulted in the presence of the pathogen only in salt and only in lettuce, respectively. Findings from other studies support our observations that the initial bacterial loads do not always correlate with the transfer levels [33, 34]. These differences are most probably related to uncontrolled experimental parameters: different volunteers, contact area, duration of contact, the force applied with fingers, randomly selection of salt particles to season the lettuce, strain variability (taking also into consideration naturally occurring *Campylobacter*), etc. Also, in the kitchen, most consumers touch chicken with their bare hands and not gloves, with different food soils (e.g., oil for marination), different pressures and different hand washing practices, so a wide variation in cross-contamination scenarios occur. Nevertheless, the present study demonstrates that cross-contamination via salt may happen and should be investigated further. These parameters were not controlled because this study was designed to mimic realistic settings. We were only able to obtain quantitative data in four positive samples (two salt and two lettuce samples), when the chicken skin was inoculated with the highest levels of *Campylobacter*. Nevertheless, the detection of *Campylobacter* in other samples even in numbers below < 10 UFC/g is worrisome as the infectious dose required for *Campylobacter* infection is thought to be low, particularly in high risk populations [35]. Further studies will be necessary to produce more quantitative microbiological data enabling estimation of the transfer rates from chicken to hands, to salt, and then to lettuce, and to determine the risk of campylobacteriosis caused by this specific transfer route. Nevertheless, proper hand-washing or prevention of hand contact with poultry must be stressed. Studies on consumer behaviour indicate there is lack of awareness and knowledge concerning the risk of hand contamination during handling and preparation of poultry at home [20].

After 6 hours of exposure to salt, electron microscopy revealed some *Campylobacter* cells exhibiting their typical spiral shape in one sample from which *Campylobacter* was unable to grow in culture media. Based on previous evidence supporting that many bacteria enter a viable but non-culturable (VBNC) state as a survival strategy upon exposure to environmental stressful conditions [36], we hypothesize that these cells are probably viable but non-culturable. Although a shift from the spiral morphology to coccoid form when *Campylobacter* cells enter in a VBNC state has been observed [37, 38], others found that non-culturability does not always relate to morphological change in *Campylobacter* cells [39–41]. The occurrence of VBNC in food is frequent and resuscitation is a worrying possibility particularly with respect to foodborne pathogens [42].

In conclusion this study documents cooking salt as a “novel” indirect cross-contamination route for *Campylobacter* spp. from raw chicken to RTE salads, through unwashed hands after handling artificially contaminated chicken parts. The detection of *Campylobacter* in salt seasoned lettuce coming from contaminated chicken skin, even at low levels, and its survival on salt over a time period that allows the preparation of a complete meal, indicates a high risk for foodborne illness. These results throw a new light on an extensively studied issue that is the complexity of transmission routes associated with domestic *Campylobacter* infection. The high occurrence of *Campylobacter* in poultry, despite intense control efforts, and the lack of mitigation technologies to effectively reduce the contamination levels in carcasses, underline the importance of consumer food-handling behaviours for the prevention of campylobacteriosis. Observational and microbiological studies, carried out in real domestic settings, are crucial to identify the most common food mishandling practices and to improve consumer-focused risk communication strategies. In addition, this study raises concerns regarding the possibility of cooking salt being a source of dissemination of foodborne illnesses and the survival of other foodborne pathogens in this matrix should be addressed in the future.

## Supporting information

S1 File Supplementary tables. (DOCX)

S1 Data (XLSX)

S2 Data (XLSX)
