# Supplementary material for: Cross-contamination of lettuce with Campylobacter spp. via cooking salt during handling raw poultry
Source: PLoS One. 2021 May 19;16(5):e0250980. doi: 10.1371/journal.pone.0250980 (PMC8133440; doi:10.1371/journal.pone.0250980)
Supplement: S1 File — (DOCX) [file pone.0250980.s001.docx]

**S1 File. Supplementary tables.**

S1 Table **Answers to the question “How likely is it that you would clean your hands immediately after touching the chicken?”**

| Possible answers | Denmark (N=816) | France (N=706) | Germany (N=652) | Greece (N=790) | Hungary (N= 921) | Norway (N=844) | Portugal (N=609) | Romania (N=894) | Spain (N=718) | United Kingdom (N=916) |
| --- | --- | --- | --- | --- | --- | --- | --- | --- | --- | --- |
| No chance or almost no chance (1 in 100) | 0.6 | 2.1 | 0.8 | 1.5 | 1.6 | 1.3 | 1.6 | 0.9 | 1.5 | 1.3 |
| Very slight possibility (1 in 10) | 1.1 | 2.0 | 1.2 | 0.9 | 1.3 | 1.4 | 1.8 | 1.2 | 1.7 | 1.4 |
| Slight possibility (2 in 10) | 1.8 | 3.8 | 1.1 | 0.9 | 2.0 | 1.2 | 1.5 | 2.9 | 2.2 | 2.9 |
| Some possibility (3 in 10) | 2.6 | 4.1 | 6.0 | 2.0 | 2.2 | 3.0 | 3.4 | 3.6 | 2.2 | 3.6 |
| Fair possibility (4 in 10) | 3.9 | 4.5 | 5.1 | 2.3 | 3.8 | 4.5 | 3.9 | 3.9 | 3.3 | 3.5 |
| Fairly good possibility (5 in 10) | 3.3 | 7.1 | 5.4 | 3.9 | 9.1 | 3.9 | 5.3 | 8.4 | 5.8 | 4.0 |
| Good possibility (6 in 10) | 1.8 | 7.2 | 5.1 | 4.6 | 5.6 | 6.8 | 5.1 | 6.5 | 7.5 | 4.7 |
| Probable (7 in 10) | 2.6 | 8.5 | 5.5 | 3.2 | 7.6 | 5.1 | 5.3 | 4.1 | 9.6 | 4.0 |
| Very probable (8 in 10) | 5.5 | 9.2 | 12.1 | 6.2 | 11.6 | 12.0 | 9.7 | 9.2 | 13.2 | 6.1 |
| Almost sure (9 in 10) | 10.4 | 13.3 | 15.2 | 17.3 | 17.5 | 8.6 | 18.6 | 18.2 | 16.0 | 11.0 |
| Certain or practically certain (99 in 100) | 66.3 | 38.1 | 42.6 | 57.2 | 37.7 | 52.3 | 43.8 | 41.1 | 36.8 | 57.3 |

S2 Table  **Answers to the question “How would you clean your hands?”**

| Possible answers | Denmark (N=816) | France (N=706) | Germany (N=652) | Greece (N=790) | Hungary (N= 921) | Norway (N=844) | Portugal (N=609) | Romania (N=894) | Spain (N=718) | United Kingdom (N=916) |
| --- | --- | --- | --- | --- | --- | --- | --- | --- | --- | --- |
| I wash my hands with cold water | 8.3 | 14.6 | 9.8 | 7.3 | 5.2 | 5.7 | 18.2 | 9.3 | 18.2 | 7.0 |
| I wash my hands with warm water | 34.7 | 29.3 | 37.9 | 28.4 | 46.1 | 40.2 | 26.3 | 44.2 | 25.2 | 33.8 |
| I wash my hands in running water | 30.1 | 35.0 | 42.9 | 20.8 | 22.9 | 33.5 | 42.0 | 28.9 | 28.0 | 32.2 |
| I wash my hands with regular soap (bar or liquid) | 67.3 | 42.5 | 46.3 | 61.0 | 36.6 | 58.9 | 41.2 | 48.0 | 47.6 | 32.1 |
| I wash my hands with antibacterial soap | 9.8 | 12.3 | 12.1 | 22.5 | 16.2 | 12.8 | 13.0 | 16.4 | 13.0 | 39.7 |
| I make sure I wash my hands for at least 20 sec | 16.5 | 14.3 | 21.0 | 18.7 | 9.6 | 17.4 | 11.0 | 14.7 | 13.9 | 21.4 |
| I dry my hands using a tea towel or cloth | 11.0 | 26.5 | 16.7 | 10.8 | 7.1 | 12.0 | 24.8 | 17.6 | 22.6 | 16.0 |
| I dry my hands using kitchen roll | 8.3 | 9.8 | 17.8 | 12.4 | 11.4 | 16.8 | 12.3 | 14.4 | 17.8 | 12.9 |
| I let my hands dry in the air | 2.2 | 4.2 | 3.8 | 1.1 | 1.3 | 3.7 | 1.8 | 2.8 | 3.5 | 3.9 |
| I disinfect my hands with a hand disinfectant | 8.3 | 4.2 | 6.1 | 11.0 | 8.8 | 5.1 | 5.4 | 4.3 | 4.7 | 7.8 |
| I don't wash my hands | 0.6 | 1.6 | 0.3 | 0.0 | 0.2 | 0.1 | 0.0 | 0.3 | 0.1 | 0.2 |
| Other | 0.9 | 0 | 0.2 | 0.8 | 0.8 | 0.6 | 1.0 | 0.1 | 0.4 | 0.4 |
| None of the above | 0.2 | 0.4 | 0.0 | 0.5 | 0.3 | 0.5 | 0.3 | 0.0 | 0.3 | 0.7 |

S3 Table **Answers to the question “Typically, do you touch the chicken with your bare hands when preparing it?”**

| Possible answers | Denmark (N=816) | France (N=706) | Germany (N=652) | Greece (N=790) | Hungary (N= 921) | Norway (N=844) | Portugal (N=609) | Romania (N=894) | Spain (N=718) | United Kingdom (N=916) |
| --- | --- | --- | --- | --- | --- | --- | --- | --- | --- | --- |
| No, I use gloves | 3.9 | 4.7 | 7.1 | 16.8 | 2.6 | 7.8 | 7.8 | 7.8 | 8.6 | 6.1 |
| No, I use a plastic bag as a glove | 2.3 | 1.8 | 2.3 | 2.9 | 1.1 | 3.6 | 1.6 | 2.1 | 2.1 | 3.8 |
| No, I avoid touching it by using kit | 1.3 | 3.4 | 4.6 | 3.2 | 1.3 | 3.9 | 1.8 | 1.6 | 2.4 | 3.7 |
| No, I use a fork | 14.0 | 15.0 | 6.9 | 3.5 | 2.9 | 11.1 | 4.8 | 4.6 | 6.3 | 7.0 |
| Yes, when cutting | 52.1 | 46.7 | 53.7 | 51.0 | 69.8 | 69.8 | 65.2 | 67.9 | 54.9 | 51.9 |
| Yes, when moving it to a bowl/pot/pa | 41.2 | 41.1 | 42.5 | 35.9 | 53.1 | 29.3 | 47.1 | 46.5 | 43.5 | 42.6 |
| Yes, when adding salt/spices/butter/ | 39.6 | 24.4 | 41.7 | 38.5 | 47.6 | 23.0 | 34.8 | 46.3 | 38.7 | 28.6 |
| Yes, when I rinse it | 36.9 | 18.3 | 46.2 | 51.9 | 60.8 | 30.8 | 53.7 | 55.7 | 27.9 | 25.8 |
| Yes, when I wipe it with kitchen rol | 19.1 | 15.2 | 29.8 | 16.5 | 15.5 | 15.9 | 9.0 | 18.6 | 15.6 | 11.2 |
| Other | 1.0 | 0.1 | 0.2 | 0.4 | 0.8 | 0.6 | 0.2 | 0.2 | 0.1 | 0.2 |
| None of the above | 1.2 | 0.6 | 0.8 | 0.3 | 0.2 | 1.2 | 0.3 | 0.4 | 0.7 | 1.7 |

S4 **Table use of salt in different countries**

| **Country** | **Participant** | **Salt shaker/bowl** | **Was it picked by hands to season the chicken?** | **Do consumers picked salt by hands when handling chicken (touching raw chicken, seasoning, opening packs) without washing hands?** | **What type of salt was normally used for seasoning salad? The same used for seasoning chicken?** |
| --- | --- | --- | --- | --- | --- |
| Hungary | HU_MÁ_N | bowl | no | no | yes |
|  | HU_JU_V | bowl | yes | no | yes |
|  | HU_ED_V | bowl | yes | yes | yes |
|  | HU_MA_B | bowl | yes | yes | yes |
|  | HU_KA_ZS | bowl | no | no | yes |
|  | HU_BA_N | shaker | no | no | yes |
|  | HU_BE_N | salt grinder | no | no | yes |
|  | HU_ÁG_V | bowl | no | no | yes |
|  | HU_NÓ_B | bowl | no | no | no (salt was not used for chicken) |
|  | HU_ÁG_ZS | bowl | no | no | yes |
|  | HU_VI_N | bowl | yes | yes | yes |
|  | HU_PA_V | no salt was used during cooking (2 spice mixtures was used) | | | |
|  | HU_ZS_B | bowl | no | no | yes |
|  | HU_FE_ZS | shaker | no | no | yes |
|  | HU_LE_ZS | bowl | yes | yes | no (salt grinder was used for vegetables) |
| Norway | Georg | Not known (Spice mix shaker) | No | No | Only used salt for the salad. |
|  | Emma | *Box | No | No | Only used for asparagus. |
|  | Inger | *Box | No | No |  |
|  | Fredrik | Bowl | Yes | No (cleaned hands with soap and water before touching salt) |  |
|  | Chris/Camilla | *Box | no | no | Chris uses his hands to salt the pasta and a pepper shaker for the frying/boiling chicken, but does not salt the salad or the raw chicken. |
|  | Oda/Ove | Shaker/salt box (original packaging) | No, salt poured into hand | No (washed their hands after touching chicken) | Washed their hands after touching chicken. |
|  | Laura | Shaker | No | No |  |
|  | Bente | Shaker | No | No, did not touch chicken | She did not salt the salad, just added some olive oil. |
|  | Nils | Bowl | No – used spoon | No – used spoon | He used table salt for seasoning the salad, pouring it into a cup with oil and vinegar as part of making the salad sauce. |
|  | Petter | Not known | No | No | Only added salt to water. Washed hands after touching chicken |
|  | Anna | Shaker | No | No | Salted salad with shaker, after thoroughly washing hands after touching chicken. |
|  | Hanne | Shaker | No | No | Sprinkles salt in young child’s (boy age 2.5) hands to salt the rice. Hanne use a shaker to salt the chicken in the frying pan, but does not salt the salad. |
|  | Jon | Unknown, but used a spice mix | No | Yes, massaged together with the chicken by hand |  |
|  | Kari | Shaker | No | No | She said they normally used table salt (and pepper) for all kinds of meals. |
|  | Roger | *Box | no | no | He never salted the chicken. He did not prepare any salad. |
| Portugal | Carlos (24 years, urban) | bowl | no | no | no (salt was not used for chicken; but picked by hand to season salad) |
|  | Bernardo (19 years, urban) | bowl | yes | no | no (salt was not used to season salad) |
|  | André (30 years, urban) | Salt was not used |  |  |  |
|  | Marta (35 years, urban) | bowl | yes | yes | yes |
|  | Vanessa (29 years, rural) | bowl | no | Yes | yes |
|  | Sónia (42 years, rural) | bowl | Yes | Yes | yes |
|  | Andreia (33 years, urban) | Automatic Shaker | No | No | She used an automatic salt shaker that grinded salt automatically while seasoning chicken at the beginning of cooking only. Not during preparation of chicken. Used the same shaker for seasoning salad, but did this after the cooking of chicken. |
|  | Filipa (36 years, urban) | bowl | Yes | Yes | yes |
|  | Sílvia (33 years, rural) | bowl | no | no | Used a spoon to season the chicken with salt only when it was at the beginning of cooking chicken (not during preparation). Did not use salt to season the salad. |
|  | Josefina (81 years, urban) | bowl | Yes | Yes | Did not prepare salad for this meal |
|  | Emília (89 years, urban) | bowl | yes | yes | Did not use salt to season this particular salad |
|  | Augusto (70 years, rural) | bowl | yes | No | Did not season the salad |
|  | Manel (73 years, urban) | bowl | Yes | Yes | yes |
|  | Odete (65 years, urban) | bowl | No | No | Salt was used to season rice and not chicken or salad. She grabbed a spoon to remove the salt from the bowl. |
|  | Celeste (70 years, urban) | bowl | yes | Yes | yes |
| Romania | Zoltan, YSM | shaker | No | No | Yes |
|  | Sorina, YF | bowl | no | yes | Yes |
|  | Fanica, E | shaker | no | no | Yes |
|  | Maria Mirabela ,YF | bowl | no | no | Yes |
|  | Damian, E | bowl | yes | yes | Yes |
|  | Amalia, YF | bowl | no | No | Yes |
|  | Dumitra, E | bowl | yes | yes | yes |
|  | Balanel, YSM | bowl | no | no | yes |
|  | Domnica, E | bowl | no | no | yes |
|  | Linalia, E | bowl | no | no | yes |
|  | Minodora, YF | bowl | yes | no | yes |
|  | Bogdan, YSM | shaker | no | no | yes |
|  | Florinel, YSM | shaker | no | no | yes |
|  | Ionel, YSM | shaker | no | no | yes |

* The boxes are made of carton and meant to refill home shakers/grinders

**S5 Table - *Campylobacter jejuni* and *Campylobacter coli* strains used in this study.**

| Isolate Code | Specie | Origin | Sample type | Geographic isolation |
| --- | --- | --- | --- | --- |
| NCTC 11168 | *C. jejuni* | Human | Faeces | NA |
| DSM 4688 | *C. jejuni* | Animal | Bovine Faeces | Belgium |
| C9 | *C. jejuni* | Food | Chicken^1^ | Portugal |
| C21A | *C. jejuni* | Food | Chicken^1^ | Portugal |
| DFVF1099 | *C. jejuni* | Food | Chicken^2^ | Denmark |
| CJ305 | *C. jejuni* | Turkey slaughterhouse | NA^3^ | Germany |
| DSM 4689 | *C. coli* | Animal | Pig Faeces | Belgium |
| C3 | *C. coli* | Food | Chicken^1^ | Portugal |

DSM - German Collection of Microorganisms and Cell Cultures GmbH, Germany;

NCTC – National Collection of Type Cultures, UK;

NA – information not available.

1 - Cardoso MJ, Ferreira V, Truninger M, Maia R, Teixeira P. Cross-contamination events of *Campylobacter* spp. in domestic kitchens associated with consumer handling practices of raw poultry. Submitt Publ Int J Foof Microbiol (FOOD-D-20-00334).

2 - Takamiya M, Ozen A, Rasmussen M, Alter T, Gilbert T, Ussery DW, et al. Genome sequences of two stress-tolerant *Campylobacter jejuni* poultry strains, 305 and DFVF1099. J Bacteriol. 2011;193(19):5546–7.

3 - Alter T, Gaull F, Froeb A, Fehlhaber K. Distribution of *Campylobacter jejuni* strains at different stages of a turkey slaughter line. Food Microbiol. 2005;22(4):345–51.
